# Association between trunk and gluteus muscle size and long jump performance

**DOI:** 10.1371/journal.pone.0225413

**Published:** 2019-11-19

**Authors:** Katsuki Takahashi, Taku Wakahara

**Affiliations:** 1 Graduate School of Health and Sports Science, Doshisha University, Kyoto, Japan; 2 Faculty of Health and Sports Science, Doshisha University, Kyoto, Japan; 3 Human Performance Laboratory, Waseda University, Saitama, Japan; University of Mississippi, UNITED STATES

## Abstract

The present study aimed to examine the sizes of trunk and gluteus muscles in long jumpers and its relation to long jump performance. Twenty-three male long jumpers (personal best record in long jump: 653–788 cm) and 22 untrained men participated in the study. T1-weighted magnetic resonance images of the trunk and hip were obtained to determine the cross-sectional areas of the rectus abdominis, internal and external obliques and transversus abdominis, psoas major, quadratus lumborum, erector spinae and multifidus, iliacus, gluteus maximus, and gluteus medius and minimus. The cross-sectional areas of individual trunk and gluteus muscles relative to body mass were significantly larger in the long jumpers than in untrained men (*P* < 0.001, Cohen’s *d* = 1.3–4.3) except for the gluteus medius and minimus. The relative cross-sectional area of the rectus abdominis of takeoff leg side was significantly correlated with their personal best record for the long jump (*r* = 0.674, corrected *P* = 0.004). Stepwise multiple regression analysis selected relative cross-sectional areas of the rectus abdominis and iliacus and the personal best record in 100-m sprint to predict the long jump distance (standard error of estimate = 22.6 cm, adjusted *R*^*2*^ = 0.763). The results of the multiple regression analysis demonstrated that the rectus abdominis and iliacus size were associated with long jump performance independently of sprint running capacity, suggesting the importance of these muscles in achieving high performance in the long jump.

## Introduction

Long jump is one of the track and field events, in which athletes compete for the horizontal jump distance. The distance in the long jump is divided into the takeoff, flight and landing distances [1]. Approximately 90% of the total jump distance is accounted for by the flight distance, which is primarily determined by the speed of the center of mass (CM) at takeoff^1^. Several studies have reported a positive correlation between the speed at takeoff and the total jump distance [1–3]. The speed consists of two components: the horizontal velocity increased through the approach phase, and the vertical velocity generated during the takeoff phase. Therefore, a fast run-up to obtain a high horizontal velocity and effective takeoff to generate a high vertical velocity while maintaining the horizontal velocity are essential for achieving high performance in long jump.

It is reasonable to expect that the horizontal velocity during the approach phase in the long jump is strongly related to the individual sprint running capacity. A previous kinetic study of sprint running indicated that the hip extension torque contributed to the generation of forward propulsion of CM [4], suggesting the importance of the hip extensor muscles. On the other hand, a high sprint speed was also attained by the hip flexion torque during the swing phase, which results in the fast leg swing and a high step frequency [5]. In fact, a negative correlation was demonstrated between the sprint time and the size of hip extensors and flexors [6,7]. Based on these findings, large hip extensors and flexors may be required for obtaining a high horizontal velocity in the approach phase of the long jump.

It has been demonstrated that the vertical velocity gain during the takeoff phase is primarily achieved during body pivoting, which is an action of the body moving over the fixed foot of the takeoff leg [3]. During the body pivoting, the vertical impact force acted to accelerate the body vertically, which could lead to a substantial gain in vertical velocity [8]. To accomplish effective pivoting, Graham-Smith and Lees [8] suggested that the leg strength, especially the hip extension and abduction strength, should be strong enough to resist the huge impact force on the body during the takeoff phase. Hence, the hip extensors and abductors may play important roles in the takeoff phase. Meanwhile, it is possible that the trunk muscles also contribute to the effective takeoff, because the force generated by these muscles can increase the stability and stiffness of the spine [9], which may be required to resist the impact force during the takeoff phase. Moreover, the trunk stability can provide a foundation for force production around the hip joint during physical movements [10,11]. For example, Tayashiki et al. [12] reported that the magnitude of the intra-abdominal pressure (IAP) was associated with the maximal voluntary hip extension torque. The IAP was demonstrated to be increased by the contraction of the abdominal muscles [13,14]. Based on these findings, the abdominal muscles can contribute to the enhancement of the hip extension torque by increasing IAP during takeoff phase.

The function of a muscle is strongly related to its architecture, such as cross-sectional area (CSA), fascicle length, pennation angle, and moment arm [15]. Muscle CSA is strongly related to the maximal force of the muscle [16,17]. Moreover, Trezise et al. [18] demonstrated that the muscle CSA was more influential than the other architectural factors (pennation angle, fascicle length and moment arm) in generating the force. Hence, large CSAs of the above-mentioned muscles could be advantageous for achieving high performance in long jump. However, large muscle CSAs entail a large body mass, having a negative effect on increasing the horizontal and vertical velocity of CM. Thus, it may be important for long jumpers to know which muscle’s CSA is associated with the long jump distance. Nevertheless, there is no study examining this association. The purpose of the present study was to explore the profile of CSA of trunk and gluteus muscles in long jumpers and its relation to long jump performance. According to the previous findings, the gluteus maximus (Gmax) is the largest muscle among the hip extensors [19], and it also acts as a hip abductor [20]. Thus, we hypothesized that 1) experienced long jumpers would have larger CSAs of the abdominal muscles and Gmax than untrained individuals, and 2) CSAs of these muscles would be correlated with long jump distance.

## Methods

### Subjects

Twenty-three male long jumpers (age: 20.8 ± 1.6 years, body height: 174.7 ± 5.2 cm, body mass: 67.0 ± 6.5 kg, years of experience: 7.4 ± 3.0 years, mean ± standard deviation [SD]) and 22 untrained men (age: 22.4 ± 1.5 years, body height: 171.8 ± 7.2 cm, body mass: 65.9 ± 5.9 kg) participated in the present study. The personal best records for the long jump and the 100-m sprint of long jumpers ranged from 653 to 788 cm (721.6 ± 46.3 cm), and 10.41 to 11.94 s (11.15 ± 0.39 s), respectively. Their personal best records for the long jump were achieved in an official competition in the same year as the testing (n = 13), the previous year (n = 6), or more than one year ago (n = 4), while those for the 100-m sprint were achieved in an official competition in the same year as the testing (n = 5), the previous year (n = 11) or more than one year ago (n = 7). Most of the long jumpers had engaged in regular resistance training programs including squat, power clean and bench press exercises (approximately one or two days per week). None of the untrained men had engaged in competitive sports or regular resistance training for at least 1 year before the test. This study was approved by the Doshisha Ethics Review Committee (16035) and conducted in accordance with the Declaration of Helsinki. All subjects were informed of the purpose and potential risk of the experiments and gave written informed consent.

### Magnetic resonance (MR) imaging

T1-weighted MR images (slice thickness: 6 mm, gap: 4 mm, echo time: 8.8 ms, repetition time: 500 ms, field of view: 360/400 mm, matrix: 256 × 192) of the trunk and hip were obtained using a 1.5-T scanner system (Echelon VEGA, Hitachi, JPN) with a 16-channel body array coil. The subject was placed in supine and prone positions in the magnet bore to obtain the trunk and hip images, respectively, with the knee and hip joints extended. Each scanning was performed during a 20-s breath-hold to prevent an influence of motion artifact caused by respiration. The long jumpers were instructed to refrain from practice and training hard in the testing day until MR imaging.

**Fig 1:**
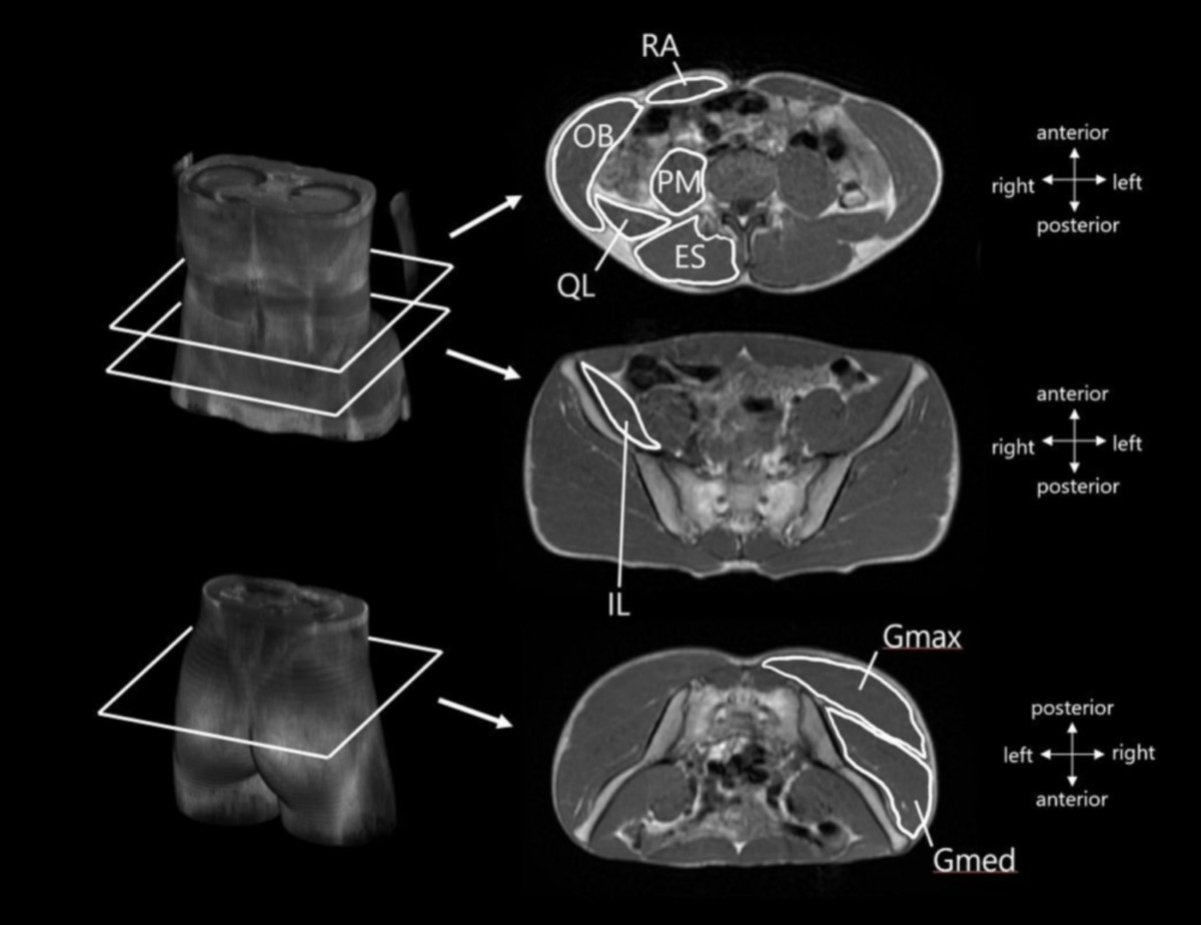
Examples of muscle cross-sectional areas (CSAs) in magnetic resonance (MR) images. RA: rectus abdominis, OB: internal and external obliques and transversus abdominis, PM: psoas major, QL: quadratus lumborum, ES: erector spinae and multifidus, IL: iliacus, Gmax: gluteus maximus, Gmed: gluteus medius and minimus.

The collected images were reconstructed to a matrix size of 512 × 512. The CSAs of the following eight muscles or muscle groups were analyzed using ImageJ software (National Institute of Health, USA) by tracing their borders: 1) rectus abdominis (RA), 2) internal and external obliques and transversus abdominis (OB), 3) psoas major (PM), 4) quadratus lumborum (QL), 5) erector spinae and multifidus (ES), 6) iliacus (IL), 7) Gmax, and 8) gluteus medius and minimus (Gmed) (Fig 1). The CSA was measured for several slices along the belly of the muscle (group) by an investigator. The slice in which the CSA was maximal was identified for each muscle (group), and then the muscle CSA in the slice was measured twice. The mean value of the two measurements of the muscle CSA was used for subsequent analysis. The CSA of each muscle (group) was determined for both takeoff and free (non-takeoff) leg sides. The coefficient of variation and intraclass correlation coefficient of two measurements of muscle CSA in the untrained men were 1.0 ± 0.8% and ≥ 0.987, respectively. Subcutaneous fat CSA was measured at the Jacoby line.

Generally, muscle size is proportional to the body mass. Meanwhile, large body mass has a negative effect on velocity of center of mass and thus long jump distance. Therefore, it is assumed that muscle size relative to body mass is associated with the long jump distance. According to allometric scaling [21], CSA is a function of length to the second power and mass is a function of length to the third power. Thus, the ratio of muscle CSA to two-thirds power of body mass was calculated as the relative muscle CSA (cm^2^/kg^2/3^) to account for the influence of body mass on muscle CSA.

### Statistical analysis

The Shapiro–Wilk test was used to check the normal distribution of the measured variables. As all variables were normally distributed, the statistical analyses were conducted by parametric tests. Unpaired *t*-tests with Bonferroni correction were conducted to test the group (long jumpers vs. untrained men) differences in relative muscle CSA (mean value of both sides) and subcutaneous fat CSA. Side-to-side asymmetry of muscle CSA in the long jumpers was assessed by paired *t*-test with Bonferroni correction. Cohen’s *d* and its 95% confidence interval (CI) were calculated as an index of effect size of group and side-to-side differences in muscle and subcutaneous fat CSA. Simple linear correlation between the relative muscle CSA of each side and the personal best record for the long jump was tested using a Pearson’s product moment correlation coefficient. The *P* values of the correlations were corrected by the false discovery rate method [22]. The threshold of the false discovery rate for statistical significance was set at < 0.05. The 95% CI for the correlations were calculated using the corrected *P* values [23]. A stepwise multiple regression analysis was performed to develop an equation for the personal best record for the long jump, using the relative CSAs of each muscle of each side, subcutaneous fat CSA, and 100-m sprint time as independent variables. The stepwise multiple regression analysis selected the explainable variables which satisfied the conditions that the variance inflation factor was less than 10, and *P* value of *β* was less than 0.05. The statistical significance for all analyses was set at *P* < 0.05. All statistical analyses were conducted by using IBM SPSS software (version 25; IBM, USA).

## Results

There were no significant differences between the long jumpers and untrained men in height (*P* = 0.123) or body mass (*P* = 0.548). The relative CSAs of individual trunk muscles and Gmax were significantly larger in the long jumpers than in untrained men (*P* < 0.001, *d* = 1.3–4.3, statistical power = 0.989–0.999, Table 1). However, no significant difference was found between the long jumpers and untrained men in relative CSA of Gmed (*P* = 0.074, *d* = 0.6, statistical power = 0.503). The relative CSA of subcutaneous fat was significantly smaller in the long jumpers than in untrained men (*P* < 0.001, *d* = −2.2, statistical power = 0.999).

**Table 1:** Comparisons of the cross-sectional areas (CSAs) of trunk and gluteus muscles and subcutaneous fat between long jumpers and untrained men.

Variables	Mean ± SD							% difference	Cohen’s *d* [95% CI: lower, upper limits]	
	long jumpers (n = 23)				untrained men (n = 22)					
Muscle CSA (cm^2^/kg^2/3^)										
RA	0.65	±	0.10	*	0.39	±	0.07	68	3.2	[2.2, 4.0]
OB	1.92	±	0.21	*	1.46	±	0.26	32	1.9	[1.2, 2.6]
PM	1.30	±	0.14	*	0.92	±	0.10	42	3.2	[2.3, 4.0]
QL	0.50	±	0.08	*	0.39	±	0.07	28	1.5	[0.8, 2.1]
ES	1.93	±	0.22	*	1.40	±	0.16	38	2.8	[1.9, 3.6]
Gmax	3.98	±	0.22	*	2.98	±	0.24	34	4.3	[3.2, 5.3]
Gmed	2.75	±	0.29		2.59	±	0.29	6	0.6	[−0.1, 1.1]
IL	0.75	±	0.09	*	0.64	±	0.08	17	1.3	[0.6, 1.9]
Subcutaneous fat CSA (cm^2^/kg^2/3^)	2.83	±	0.57	*	6.01	±	1.95	−53	−2.2	[−2.9, −1.5]

* Significant difference in muscle size between long jumpers and untrained men.

Relative muscle CSAs are mean value of each leg side. % difference = (CSA_1_ –CSA_u_) / CSA_u_ × 100. CSA_l_: group mean value of relative CSA in long jumpers, CSA_u_: group mean value of relative CSA in untrained men, SD: standard deviation, CI: confidence interval, RA: rectus abdominis, OB: internal and external obliques and transversus abdominis, PM: psoas major, QL: quadratus lumborum, ES: erector spinae and multifidus, Gmax: gluteus maximus, Gmed: gluteus medius and minimus, IL: iliacus.

There was a significant side-to-side difference in CSA of RA. The CSA of RA of the takeoff leg side was significantly greater than that of the free leg side (*P* < 0.001, *d* = 0.3, statistical power = 0.280, Table 2). The side-to-side differences were not significant in CSAs of the other muscles (*P* = 0.175–0.847, *d* ≤ 0.2, statistical power ≤ 0.151).

**Table 2:** Comparisons of the cross-sectional areas (CSAs) of trunk and gluteus muscles between takeoff and free leg sides in long jumpers.

Variables	Mean ± SD							% difference	Cohen’s *d* [95% CI: lower, upper limits]	
	takeoff leg side				free leg side					
Muscle CSA (cm^2^)										
RA	11.1	±	2.0	*	10.5	±	2.0	5.4	0.3	[−0.3, 0.9]
OB	31.6	±	3.9		31.9	±	4.6	−1.0	0.1	[−0.5, 0.7]
PM	21.6	±	3.1		21.5	±	2.8	0.3	0.0	[−0.6, 0.6]
QL	8.2	±	1.6		8.1	±	1.3	0.8	0.0	[−0.5, 0.6]
ES	31.8	±	4.9		32.0	±	4.8	−0.8	0.1	[−0.5, 0.6]
Gmax	66.0	±	6.5		65.5	±	7.3	0.8	0.1	[−0.5, 0.7]
Gmed	45.2	±	6.6		45.4	±	5.9	−0.4	0.0	[−0.5, 0.6]
IL	12.1	±	1.7		12.5	±	1.8	−3.2	0.2	[−0.4, 0.8]

* Significant difference in muscle size between takeoff leg and free leg sides.

% difference = (CSA_t_—CSA_f_) / CSA_f_ × 100. CSA_t_: group mean value of CSA of takeoff leg side, CSA_f_: group mean value of CSA of free leg side, SD: standard deviation, CI: confidence interval, RA: rectus abdominis, OB: internal and external obliques and transversus abdominis, PM: psoas major, QL: quadratus lumborum, ES: erector spinae and multifidus, Gmax: gluteus maximus, Gmed: gluteus medius and minimus, IL: iliacus.

**Fig 2:**
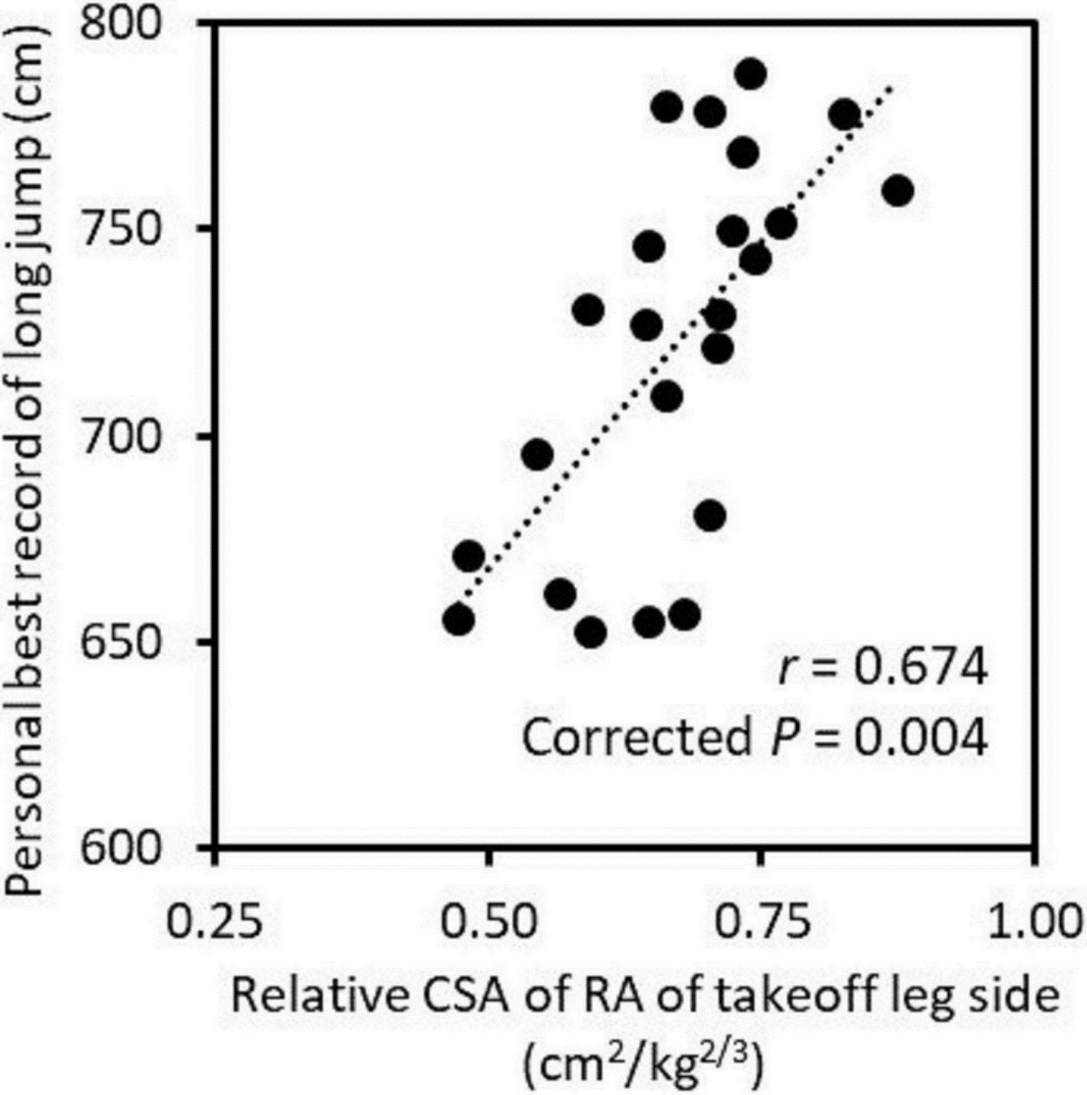
The relationship between the relative cross-sectional area (CSA) of the rectus abdominis (RA) of takeoff leg side and personal best record for the long jump. The *P* value was corrected by the false discovery rate method [22].

The relative CSA of RA of takeoff leg side was significantly correlated with the personal best record for the long jump (*r* = 0.674, corrected *P* = 0.004, statistical power = 0.962, Fig 2). There was also a significant correlation between 100-m sprint time and personal best record for the long jump (*r* = −0.719, corrected *P* = 0.002, statistical power = 0.985). However, no significant correlation was found between the relative CSAs of the other muscle or subcutaneous fat CSA and personal best record for the long jump (*r* = 0.004–0.490, corrected *P* = 0.094–0.985, statistical power = 0.050–0.688, Table 3).

**Table 3:** Simple correlation coefficients of the cross-sectional areas (CSAs) of trunk and gluteus muscles and subcutaneous fat and 100-m sprint time with long jump distance.

Variables	Correlation r [95% CI: lower, upper limits]			
	takeoff leg side		free leg side	
Muscle CSA (cm^2^/kg^2/3^)				
RA	0.674	[0.259, 0.879] *	0.490	[−0.114, 0.829]
OB	0.129	[−0414, 0.605]	0.224	[−0.393, 0.702]
PM	0.099	[−0.420, 0.569]	0.184	[−0.391, 0.656]
QL	0.179	[−0.355, 0.625]	0.065	[−0.416, 0.517]
ES	0.255	[−0.420, 0.749]	0.261	[−0.477, 0.783]
Gmax	0.272	[−0.528, 0.816]	0.272	[−0.669, 0.878]
Gmed	0.216	[−0.364, 0.675]	0.236	[−0.416, 0.728]
IL	0.478	[−0.089, 0.811]	0.434	[−0.148, 0.793]
Subcutaneous fat CSA (cm^2^)	0.004		[−0.369, 0.376]	
100-m sprint time (s)	−0.719		[−0.901, −0.322] *	

* Significant correlation of muscle CSA and 100-m sprint time with the personal best record for long jump corrected by a false discovery rate [22] less than 0.05.

95% confidence interval (CI) was adjusted using the corrected *P* value. RA: rectus abdominis, OB: internal and external obliques and transversus abdominis, PM: psoas major, QL: quadratus lumborum, ES: erector spinae and multifidus, Gmax: gluteus maximus, Gmed: gluteus medius and minimus, IL: iliacus.

**Fig 3:**
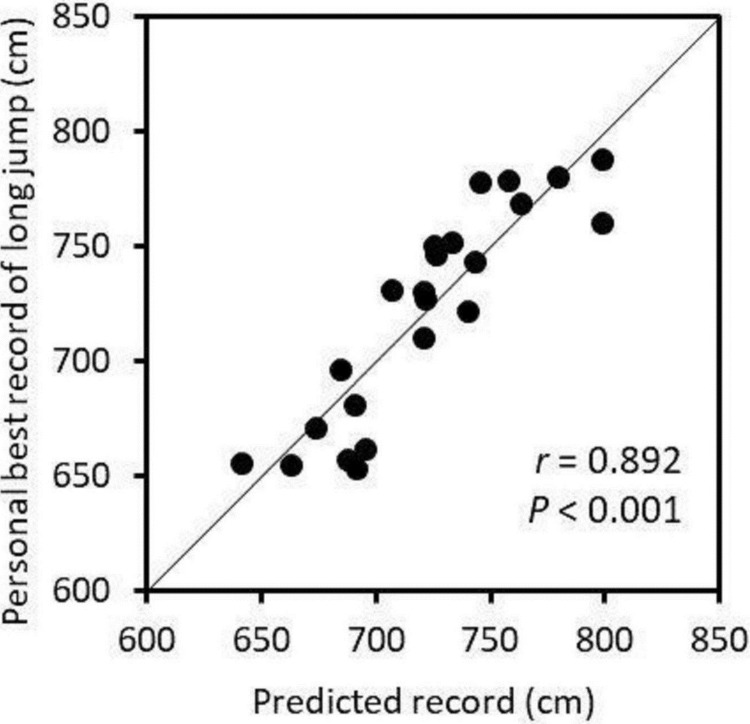
The relationship between the predicted and personal best records of the long jump. The predicted record was obtained via stepwise multiple regression analysis using the personal best record for 100-m sprint and the relative cross-sectional areas of the rectus abdominis and iliacus of takeoff leg side (standard error of estimate = 22.6 cm, adjusted *R*^*2*^ = 0.763). The solid line is an identical line.

In the stepwise multiple regression analysis, the 100-m sprint time and the relative CSAs of RA and IL of takeoff leg side were selected as the explainable variables for the personal best record for the long jump. However, the relative CSAs of the other muscles or subcutaneous fat CSA were not selected as the explainable variables (*P* = 0.146–0.927). The multiple regression analysis developed the following equation for the personal best record of the long jump: *Y* = −64.4*X*_*1*_ + 194.0*X*_*2*_ + 122.9*X*_*3*_ + 1219.6where *Y* is the personal best record for the long jump in cm, *X*_*1*_ is 100-m sprint time in s (*ß* = −0.542, *P* < 0.001), *X*_*2*_ is the relative CSA of RA of takeoff leg side in cm^2^/kg^2/3^ (*ß* = 0.411, *P* = 0.002) and *X*_*3*_ is the relative CSA of IL of takeoff leg side in cm^2^/kg^2/3^ (*ß* = 0.267, *P* = 0.025). The standard error of estimate, adjusted *R*^*2*^ and statistical power for this model were 22.6 cm, 0.763 and 0.517, respectively. When only the 100-m sprint time was used in the regression equation, *R*^2^ was 0.517 (*P* < 0.001). When the relative CSA of RA of takeoff leg side was added after 100-m sprint time, the *R*^2^ change was 0.214 (*P* = 0.001). The *R*^2^ change was 0.064 (*P* = 0.025) when the relative CSA of IL of takeoff leg side was added after the 100-m sprint time and relative CSA of RA of takeoff leg side. Fig 3 shows the relationship between the predicted and personal best records in the long jump.

## Discussion

The present results demonstrated that the relative CSAs of individual trunk muscles and Gmax were larger in the long jumpers than in untrained men. In addition, the relative CSA of RA of takeoff leg side was significantly correlated with long jump performance. Furthermore, the multiple regression analysis revealed that the relative CSAs of RA and IL of takeoff leg side and 100-m sprint time could explain 76.3% of the variability of the personal best record for the long jump. These results partly support our hypotheses that CSAs of the abdominal muscles and Gmax would be larger in long jumpers than in untrained individuals and correlated with the long jump distance. To the best of our knowledge, no study has examined the association between size of trunk and gluteus muscles and long jump performance. Although the present study is a cross-sectional study and thus cannot determine the causality of the relationships, the results suggest that large CSAs of RA and IL among the trunk and gluteus muscles may be advantageous to achieve high performance for the long jump.

**Fig 4:**
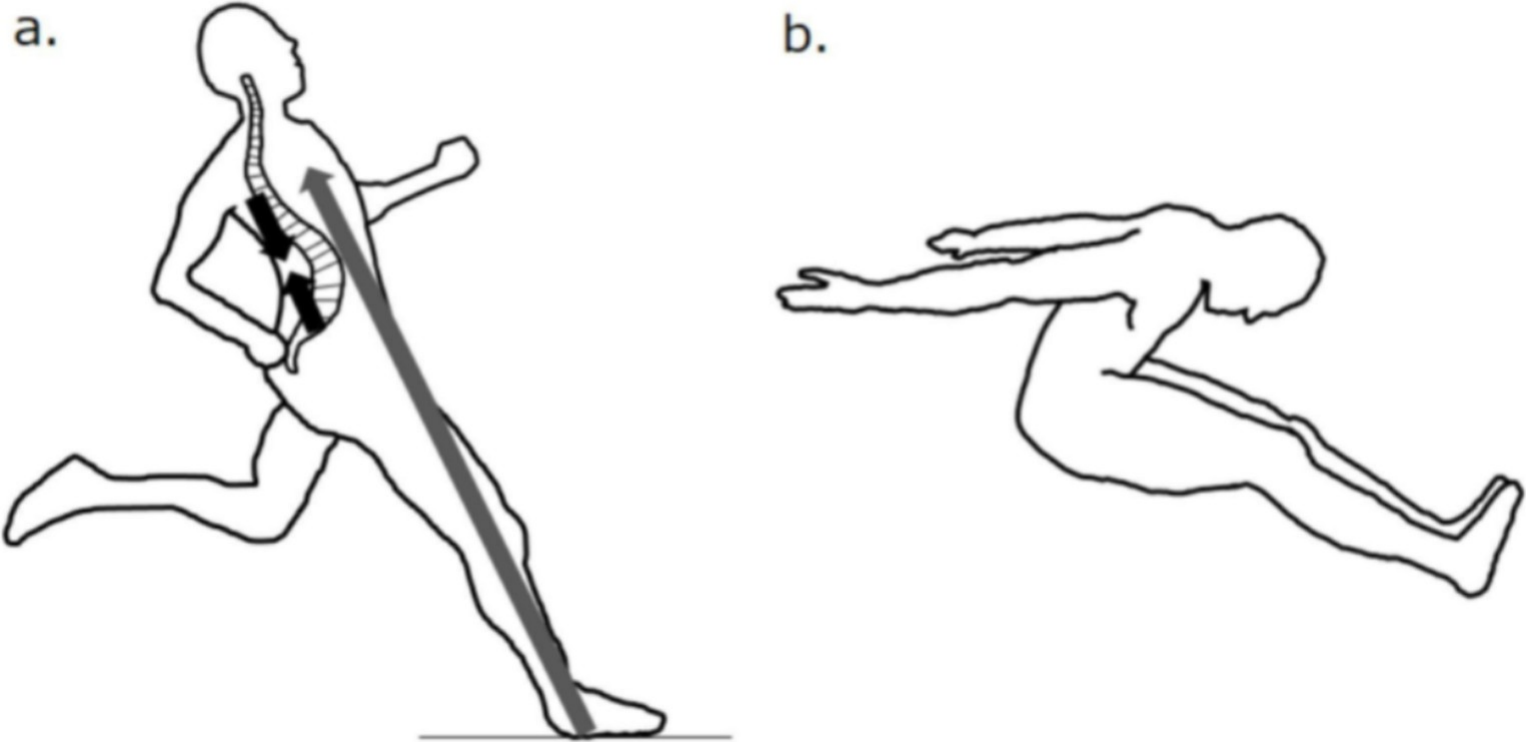
**Schematic illustrations of the body during takeoff (a) and the optimal landing position (b).** a: grey and dark arrows indicate the line of action of ground reaction force and the compression of the lumbar spine along its long axis, respectively. b: the optimal landing position is one with the hips fully flexed and the trunk well forward over the legs [25].

The relative CSAs of the abdominal muscles (RA and OB) were larger in long jumpers than in untrained men. Notably, the difference was substantially large (68%, *d* = 3.2) in RA, and the relative CSA of RA of takeoff leg side was selected as an explainable variable for the long jump distance independently of 100-m sprint time in the multiple regression analysis. These findings indicate that RA is important in the trunk motion specific to the long jump, probably in the takeoff phase, rather than the approach phase. It has been suggested that maintaining the trunk in a straight position along the line of takeoff leg was beneficial for effective pivoting in the takeoff phase [3,8]. However, this posture can lead to the compression of the lumbar spine along its long axis and result in the excessive lordosis, because the line of the trunk is nearly parallel to the line of action of ground reaction force (Fig 4A). As RA has a long moment arm of the lumbar spine flexion [24], it may contribute to the maintenance of trunk posture by resisting the lumbar spine lordosis during the body pivoting. In addition, it was demonstrated that the contraction of the abdominal muscles increased the magnitude of IAP [13,14]. The magnitude of IAP was reported to be associated with the maximal voluntary hip extension torque [12]. Therefore, it is possible that RA has the role of enhancing the hip extension torque by increasing IAP during the takeoff phase. Meanwhile, it has been suggested that the optimum landing position was one with the hips fully flexed and the trunk well forward over the legs (Fig 4B) [25]. To obtain this position, RA may also contribute to the trunk motion during the flight phase.

The present result demonstrated that the relative CSA of Gmax was larger in the long jumpers than in the untrained men. This is in line with the previous studies that reported the importance of hip extensors for long jump performance [8,26]. However, no association was found between the relative CSA of Gmax and long jump performance. One possible reason for the lack of correlation is the contribution of the other synergistic muscles (e.g., hamstrings and adductors) to the hip extension torque than Gmax. Therefore, the long jump performance could not be explained only by CSA of Gmax.

The relative CSA of IL of takeoff leg side was selected as an explainable variable for the long jump distance, suggesting the importance of IL in the long jump. In the later phase of takeoff, the hip flexion torque was exerted in the takeoff leg [26,27]. This hip flexion torque may be generated by IL of the takeoff leg. Furthermore, as mentioned above, the hip flexion was reported to be required for the optimal landing position [25]. Hence, IL may also contribute to the effective landing. Although PM is one of the agonists of hip flexion, its CSA did not correlate with the long jump performance. The reason for this result is unclear, but might be related to the different origins of PM and IL (lumbar spine vs. pelvis) and/or different activities of the two muscles depending on the motor tasks [28]. It is likely that IL, as compared to PM, would be necessary for achieving better performance in long jump.

A strong correlation was found between 100-m sprint time and long jump performance. This result is in accordance with the several previous studies [1,3]. However, no significant correlation was found between 100-m sprint time and the relative CSA of Gmax (*r* = −0.088, *P* = 0.689) or PM (*r* = −0.104, *P* = 0.636) of takeoff leg side in the present study. This result is inconsistent with the previous studies which reported the association between Gmax and PM size and the sprint running capacity [6,7]. This discrepancy between the present and previous results could be partly explained by the methodological differences (measurements of muscle size, subjects, and/or index of sprinting capacity). For example, Sugisaki et al. [7] showed the association between muscle volume of Gmax and 100-m sprint time in sprinters. Copaver et al. [6] demonstrated the association between CSA of PM and the measured time of 50-m and 120-m sprinting in athletes of various sports. However, the present study investigated the association between CSAs of Gmax and PM and 100-m sprint time in long jumpers. These differences may be related to the lack of significant correlation between CSA of Gmax and PM and 100-m sprint time.

The present study has several limitations. First, although we determined CSAs of the trunk and gluteus muscles, muscle volume was suggested to be more closely related to joint torque than CSA [29]. In addition, our measurement of muscle size was limited to the trunk and gluteus muscles due to time constraint of MR recording, whereas several studies reported that the knee and ankle joints could also contribute to the effective takeoff [3,8,26]. Therefore, the size of the muscles around the knee and ankle joints may be associated with long jump performance. Further studies should investigate the correlation between the volumes of thigh and leg muscles and long jump performance. Second, the index of sprinting and long jump performance used in the present study was the personal best record. Hence, the measured CSAs may not represent the muscle sizes at the time when the jumpers recorded their personal best records of 100-m sprint and long jump. However, although four of the long jumpers had not recorded the seasonal best time of 100-m sprint (they had not participated in an official competition for 100-m sprint in the same season as the muscle CSA were measured), the personal best record was strongly correlated with the seasonal best record for 100-m sprint time (*r* = 0.910, *P* < 0.001, n = 19) and long jump (*r* = 0.961, *P* < 0.001, n = 23), respectively. In addition, the magnitude of difference between the two records was quite small for 100-m sprint (1.4 ± 1.4%) and long jump (1.2 ± 1.8%). Furthermore, the relative CSAs of RA (*r* = 0.674, *P* < 0.001) and IL (*r* = 0.413, *P* = 0.050) of takeoff leg side were also correlated with the seasonal best record of long jump. Therefore, the time differences between the measurements of CSA, 100-m sprint time and long jump distance would not have a significant influence on the present findings. Thirdly, we used the 100-m sprint time as an index of the approach velocity of long jump, although the length of sprint approach to long jump is shorter than 100-m. Actually, the number of steps in the approach of long jump was reported to be smaller (mean: 18.5 steps [2]) than 100-m sprint (mean: 45.7 steps [30]). Thus, the 100-m sprint time cannot perfectly reflect the approach velocity of long jump. However, it was reported that the 100-m sprint time was strongly correlated with the maximal sprint velocity [31]. The sprint velocity was appeared to reach its near maximum at around the 18th step [32]. Therefore, the difference between 100-m sprint and the approach of long jump may not have a substantial influence on the main findings of the present study. Fourthly, we normalized the muscle CSA with two-thirds power of body mass, according to allometric scaling [21]. Meanwhile, from a biomechanical point of view, flight distance is determined by takeoff velocity (*v*). The velocity can be attributed to force (*F*) relative to body mass (*m*) and time (*t*); i.e. *v* = *F*/*m*×*t* (according to the relation between momentum [*mv*] and impulse [*Ft*]: *mv*’–*mv* = *Ft*). Thus, normalizing the muscle CSA with body mass (cm^2^/kg) may also provide biomechanically meaningful information. Therefore, we performed the additional analyses by using the muscle CSA normalized to body mass. As a result, findings for the comparison between long jumpers and untrained men, and the association between muscle CSA and long jump distance were similar to those observed by using muscle CSA normalized to two-thirds power of body mass (see S1 File). Thus, the difference between the normalizing methods (cm^2^/kg^2/3^ vs. cm^2^/kg) may not have a significant influence on the present findings. Lastly, we did not obtain the direct evidence linking RA or IL size to the kinematic or kinetic factors of long jump performance. Thus, it is unclear how these muscles contribute to long jump performance. Further studies are needed to investigate the association between the muscle size and the trunk, pelvis and hip motions specific to long jump.

### Practical applications

Because large muscles introduce a trade-off between high strength capacity and a large body mass, it can be important to clarify the association between size of the individual muscles and long jump performance. The present results demonstrated that 1) the CSAs of individual trunk muscles and Gmax normalized to body mass were larger in the long jumpers than in the untrained men, and 2) CSAs of RA and IL normalized to body mass were associated with long jump performance independently of sprint running capacity. This suggests that large RA and IL could be advantageous to achieve high performance in long jump. Thus, selective hypertrophy of the RA and IL among the trunk and gluteus muscles as well as improvement of sprinting capacity may be beneficial to enhance the long jump performance. However, the present study is a cross-sectional study, and thus cannot determine the causality of the observed relationship. For example, there is a possibility that large CSAs of these muscles could be a by-product of repeated stresses to specific muscle(s) during long jump rather than hypertrophy for improving the long jump performance. Moreover, the relation between an increase in muscle size and improvement of the strength is still a matter of debate [33,34]. Further study is needed to clarify the effect of muscle hypertrophy on the long jump performance.

## Conclusions

We investigated the sizes of trunk and gluteus muscles in long jumpers and its relation to long jump performance. From the present results, the long jumpers have larger Gmax and individual trunk muscles compared to those in untrained men. Moreover, the result of the multiple regression analysis indicates that the size of RA and IL is associated with long jump performance independently of sprinting capacity. This suggests that the size of RA and IL is important for achieving high performance in the long jump.

## Supporting information

S1 Raw data Physical characteristics and cross-sectional areas of muscle and subcutaneous fat in each subject. (XLSX)

S1 File Additional results. (DOCX)
