# Supplementary material for: Association between trunk and gluteus muscle size and long jump performance
Source: PLoS One. 2019 Nov 19;14(11):e0225413. doi: 10.1371/journal.pone.0225413 (PMC6863557; doi:10.1371/journal.pone.0225413)
Supplement: S1 File — (DOCX) [file pone.0225413.s002.docx]

# Additional results

The CSAs of individual trunk muscles and Gmax normalized to body mass (cm^2^/kg) were significantly larger in the long jumpers than in untrained men (*P* < 0.001, *d* = 1.2–4.0, statistical power = 0.976–0.999, Table A). However, no significant difference was found between the long jumpers and untrained men in the normalized CSA of Gmed (*P* = 0.110, *d* = 0.5, statistical power = 0.374). The CSA of subcutaneous fat normalized to body mass was significantly smaller in the long jumpers than in untrained men (*P* < 0.001, *d* = −2.3, statistical power = 0.999).

**Table A.** **Comparisons of** **the cross-sectional areas (CSAs) of trunk and gluteus muscles and subcutaneous fat normalized to body mass between long jumpers and untrained men.**

| Variables | Mean ± SD | | | | | | | % difference | Cohen’s *d*  [95% CI: lower, upper limits] | |
| --- | --- | --- | --- | --- | --- | --- | --- | --- | --- | --- |
|  | long jumpers  (n = 23) | | | | untrained men  (n = 22) | | |  |  |  |
| Muscle CSA (cm^2^/kg) |  |  |  |  |  |  |  |  |  |  |
| RA | 0.16 | ± | 0.02 | * | 0.10 | ± | 0.02 | 67 | 3.2 | [2.2, 4.0] |
| OB | 0.47 | ± | 0.05 | * | 0.36 | ± | 0.06 | 31 | 1.9 | [1.2, 2.6] |
| PM | 0.32 | ± | 0.03 | * | 0.23 | ± | 0.02 | 42 | 3.4 | [2.4, 4.2] |
| QL | 0.12 | ± | 0.02 | * | 0.10 | ± | 0.02 | 27 | 1.5 | [0.8, 2.1] |
| ES | 0.48 | ± | 0.05 | * | 0.35 | ± | 0.04 | 37 | 2.9 | [2.0, 3.7] |
| Gmax | 0.98 | ± | 0.05 | * | 0.74 | ± | 0.07 | 33 | 4.0 | [2.9, 4.9] |
| Gmed | 0.68 | ± | 0.07 |  | 0.64 | ± | 0.07 | 5 | 0.5 | [−0.1, 1.1] |
| IL | 0.18 | ± | 0.02 | * | 0.16 | ± | 0.02 | 16 | 1.2 | [0.5, 1.8] |
| Subcutaneous fat CSA (cm^2^/kg) | 0.70 | ± | 0.13 | * | 1.49 | ± | 0.48 | −53 | −2.3 | [−3.0, −1.5] |

* Significant difference in CSA between long jumpers and untrained men.

Muscle CSAs normalized to body mass are mean value of each leg side.

$\text{\% difference = (}\text{CSA}\text{l}\text{ }\text{-}\text{ }\text{CSA}\text{u}\text{) ∕ }\text{CSA}\text{u}\text{ }\text{× 100 }$

CSA_l_: group mean value of CSA normalized to body mass in long jumpers, CSA_u_: group mean value of CSA normalized to body mass in untrained men, SD: standard deviation, CI: confidence interval, RA: rectus abdominis, OB: internal and external obliques and transversus abdominis, PM: psoas major, QL: quadratus lumborum, ES: erector spinae and multifidus, Gmax: gluteus maximus, Gmed: gluteus medius and minimus, IL: iliacus.

For each side, the CSAs of RA normalized to body mass (cm^2^/kg) were significantly correlated with the personal best record for the long jump (*r* = 0.734, corrected *P* = 0.001 for takeoff leg side; *r* = 0.554, corrected *P* = 0.049 for free leg side, Table B). However, no significant correlation was found between the CSAs of the other muscles normalized to body mass and personal best record for the long jump (*r* = 0.105–0.485, corrected *P* = 0.102–0.634).

**Table B. Simple correlation coefficients of the cross-sectional areas (CSAs) of trunk and gluteus muscles normalized to body mass with long jump distance.**

| Muscle CSA (cm^2^/kg) | Correlation r [95% CI: lower, upper limits] | | | |
| --- | --- | --- | --- | --- |
|  | takeoff leg side | | free leg side | |
| RA | 0.734 | [0.361, 0.905] * | 0.554 | [0.002, 0.847] * |
| OB | 0.180 | [−0.293, 0.582] | 0.258 | [−0.302, 0.686] |
| PM | 0.137 | [−0.312, 0.536] | 0.212 | [−0.280, 0.615] |
| QL | 0.217 | [−0.304, 0.638] | 0.105 | [−0.303, 0.480] |
| ES | 0.316 | [−0.259, 0.726] | 0.322 | [−0.292, 0.748] |
| Gmax | 0.326 | [−0.340, 0.774] | 0.367 | [−0.288, 0.788] |
| Gmed | 0.251 | [−0.280, 0.664] | 0.286 | [−0.285, 0.707] |
| IL | 0.485 | [−0.105, 0.822] | 0.463 | [−0.103, 0.803] |

* Significant correlation between muscle CSA and the personal best record for long jump corrected by a false discovery rate [22] less than 0.05.

95% confidence interval (CI) was adjusted using the corrected *P* value.

RA: rectus abdominis, OB: internal and external obliques and transversus abdominis, PM: psoas major, QL: quadratus lumborum, ES: erector spinae and multifidus, Gmax: gluteus maximus, Gmed: gluteus medius and minimus, IL: iliacus.

A stepwise multiple regression analysis was performed to develop an equation for the personal best record for the long jump, using CSAs of each muscle of each side normalized to body mass (cm^2^/kg), subcutaneous fat CSA and 100-m sprint time as independent variables. As a result, the 100-m sprint time and normalized CSAs of RA and IL of takeoff leg side were selected as the explainable variables for the personal best record for the long jump. However, normalized CSAs of the other muscles or subcutaneous fat CSA were not selected as the explainable variables for the long jump distance (*P* = 0.276–0.838). The following equation was developed for the personal best record of the long jump:
*Y* = −61.1*X_1_* + 931.8*X_2_* + 411.7*X_3_* + 1174.6
where *Y* is the personal best record for the long jump in cm, *X_1_* is 100-m sprint time in s (*ß* = −0.514, *P* < 0.001), *X_2_* is normalized CSA of RA of takeoff leg side in cm^2^/kg (*ß* = 0.470, *P* < 0.001), and *X_3_* is normalized CSA of IL of takeoff leg side in cm^2^/kg (*ß* = 0.236, *P* = 0.031). The standard error of estimate, adjusted *R^2^* and statistical power for this model were 20.6 cm, 0.803 and 0.769, respectively.
